# Regionalization of intestinal microbiota and metabolites in the small intestine of the Bactrian camel

**DOI:** 10.3389/fimmu.2024.1464664

**Published:** 2024-11-26

**Authors:** Yujiao Cheng, Yan Ren, Wangdong Zhang, Jia Lu, Fei Xie, Ying-Dong Fang, Xiping Fan, Wanhong He, Wenhui Wang

**Affiliations:** ^1^ College of Veterinary Medicine, Gansu Agricultural University, Lanzhou, Gansu, China; ^2^ Davies Livestock Research Centre, School of Animal and Veterinary Sciences, University of Adelaide, Roseworthy, SA, Australia

**Keywords:** Bactrian camels, intestinal microbiota, metabolites, Peyer’s patches, intestinal regional immunity

## Abstract

**Introduction:**

Peyer's patches (PPs) are crucial antigen-inductive sites of intestinal mucosal immunity. Prior research indicated that, in contrast to other ruminants, PPs in the small intestine of Bactrian camels are found in the duodenum, jejunum, and ileum and display polymorphism. Using this information, we analyzed the microbial and metabolic characteristics in various segments of the Bactrian camel's small intestine to further elucidate how the immune system varies across different regions.

**Methods:**

In this study, the microbiota and metabolite of 36 intestinal mucosal samples, including duodenal (D-PPs), jejunal (J-PPs), and ileal PPs (I-PPs), were profiled for six Bactrian camels using 16S rRNA gene sequencing and liquid chromatography with tandem mass spectrometry (LC-MS/MS). To confirm meaningful associations, we conducted connection analyses on the significantly different objects identified in each group's results. ELISA was used to analyze the levels of IgA, IgG, and IgM in the same tissues.

**Results:**

The microbiota and metabolite profiles of J-PPs and I-PPs were found to be similar, whereas those of D-PPs were more distinct. In J-PPs and I-PPs, the dominant bacterial genera included *Clostridium*, *Turicibacter*, and *Shigella*. In contrast, D-PPs had a significant increase in the abundance of *Prevotella*, *Fibrobacter*, and *Succinobacter*. Regarding the metabolomics, D-PPs exhibited high levels of polypeptides, acetylcholine, and histamine. On the other hand, J-PPs and I-PPs were characterized by an enrichment of free amino acids, such as L-arginine, L-glutamic acid, and L-serine. These metabolic differences mainly involve amino acid production and metabolic processes. Furthermore, the distribution of intestinal immunoglobulins highlighted the specificity of D-PPs. Our results indicated that proinflammatory microbes and metabolites were significantly enriched in D-PPs. In contrast, J-PPs and I-PPs contained substances that more effectively enhance immune responses, as evidenced by the differential distribution of IgA, IgG, and IgM.

**Discussion:**

The intestinal microenvironment of Bactrian camels displays distinct regional disparities, which we propose are associated with variations in immunological function throughout different segments of the small intestine. This study highlights the specific traits of the intestinal microbiota and metabolites in Bactrian camels, offering a valuable reference for understanding the relationship between regional intestinal immunity and the general health and disease of the host.

## 1 Introduction

The gut, housing a diverse array of immune cells with unique properties, is the largest immunological organ in both humans and animals. The gastrointestinal system relies on several immune compartments to effectively respond to the numerous antigens and stimuli it encounters (1). The mesenteric lymph nodes and gut-associated lymphoid tissue, as induction sites, play a crucial role in initiating immune responses by capturing, processing, and presenting antigens. Peyer’s patches (PPs) serve as a prime example of this function. Intestinal lamina propria and epithelial cells serve as effector sites, where different types of immune cells are responsible for clearing antigens to maintain the integrity of the barrier. Trillions of bacteria reside in the gut and are essential for enhancing nutrient absorption and energy metabolism (2, 3), maintaining a healthy immune system (4), and preventing or treating intestinal diseases (5). The intestinal mucosal immune system safeguards the body against pathogens and ensures the ability to tolerate commensal microbes through the utilization of specialized recognition systems for both innate and adaptive immune responses. Recent research has emphasized the segmental distribution characteristics of the immune system in the intestines, such as lymph nodes, dendritic cells, intestinal epithelial cells, T helper 17 cells (Th17) and regulatory T cells (Treg), and antibody production (6–9). The placement of these components in different sections of the gut is strategic in order to meet the specific immunological needs of each area. Both immune cells and symbiotic microorganisms in the body exhibit similar regional distribution patterns. The variations of the physical and chemical environment throughout different sections of the gut leads to different composition of bacteria (10). The small intestine harbors a more extensive variety of microbial diversity compared to the distal intestine (11). Moreover, the microbial populations of the small intestine can be distinguished in the duodenum, jejunum, and ileum (12–14). The gut microbiota has a direct impact on gastrointestinal function and controls gastrointestinal physiology by producing various metabolites (15). Metabolites such as amino acids, polyamine compounds, short chain fatty acids, and aromatic metabolites are play a role in regulating the local immunity of the intestine (16). Studies suggest that microbial activity plays a significant role in shaping the metabolic status of various ecological niches across the gastrointestinal system, leading to the development of unique metabolic profiles in different parts (17). Furthermore, alterations in gut microbiota have been associated with a range of immunological diseases, such as inflammatory bowel disease (18), diabetes (19), and atopic disorders (20). Exploring the regional characteristics of gut microbiota can enhances our understanding of the links between host physiological processes and disease development.

Bactrian camels (*Camelus bactrianus*) are large even-toed ungulates primarily found in China and other Central Asian regions. It has two distinct subspecies, including the domestic Bactrian camel (*Camelus bactrianus*) and the wild Bactrian camel (*Camelus ferus*). They can well adapt to the tough living conditions of deserts and cold locations. Camels exhibit greater resilience to specific viral illnesses and environmental stresses compared to other species inhabiting the same geographical area, owing to their distinctive immune traits (21). Our earlier research showed that camels have a unique area of lymph aggregation in the abomasum, which is potentially associated with its distinct immunological attributes Although, this area has similar protein expression profile with ileal Peyer’s patches (22), there are variations in the microbial communities (23). Intestinal location affects the expression pattern of PPs genes, with significantly lower expression in the duodenum compared to the jejunum and ileum (24). Consequently, the distribution of PPs in the duodenum is infrequently documented in both humans and animals. However, our study showed that PPs are present in all sections of the Bactrian camel’s small intestine, with variations in morphology and distribution density. In the duodenum, jejunum, and ileum, PPs exhibit honeycomb, nodular, and saccular structures, respectively (25). This diversity may indicate regional differences in immune function within the small intestine. In PPs, a considerable proportion of lymphocytes undergo differentiation and activation, playing a pivotal role in both regulating the proliferation of symbiotic bacteria (26) and producing a range of antibodies (27). These antibodies effectively neutralize numerous antigens, thereby establishing a secondary defense line within the intestinal immune system. In addition, the gut microbiota and its metabolites exert intricate influences on adaptive immunity, such as regulating the production of IgA, IgM, and IgG (23). These findings suggest that the distribution patterns of PPs in the gut of the Bactrian camel may be related to changes in regional immunological functions. The aforementioned research indicates the possibility of investigating the variations in the immune response of Bactrian camels’ intestinal mucosal inductive sites.

To accomplish this objective, this study examined the relationships between the intestinal commensal microbiota, metabolites, and the host. Specifically, we obtained and compared the microbiota and metabolic profiles of the PPs in duodenal (D-PPs), jejunal (J-PPs), and ileum (I-PPs). On the other hand, the expression of IgA, IgG, and IgM was determined using an enzyme-linked immunosorbent assay (ELISA) for a mucosal immune characterization. It is expected to further comprehend the feature of small intestine segmental mucosal immunity in Bactrian camels and investigate the correlation between colonizing bacteria and metabolites. Moreover, the study can provide valuable references for the feeding tube and disease prevention strategies in Bactrian camels.

## 2 Materials and methods

### 2.1 Sample collection

Six healthy Bactrian camels from Minqin County in Gansu Province, China, were anaesthetized intravenously with sodium pentobarbital (20 mg/kg) and exsanguinated until death. Samples were obtained from the D-PPs, J-PPs, and I-PPs through the opening of the abdominal cavity. After opening the intestinal cavity, residual food is gently rinsed away with a sterile saline solution. Next, gently dip the mucus with a sterile cotton swab, moving from the center towards the edge of the PPs. The sample was put into a 2.5mL frozen storage tube and promptly frozen in liquid nitrogen for further use. A total of 36 samples of intestinal mucus were collected, with 12 samples each from groups D-PPs, J-PPs, and I-PPs. Out of these, a total of 18 samples (6 samples from each group) were used for microbiome analysis, while the remaining 18 samples (6 samples from each group) were used for subsequent metabolome analysis. Afterwards, tissue samples from PPs were taken for ELISA at the same time. The PPs tissues from the swab-dipped areas were cut and put into 2.5-ml frozen tubes, which were then promptly frozen in liquid nitrogen for further use.

### 2.2 Small intestine mucus microbiome analysis

#### 2.2.1 DNA extraction and sequencing

The OMEGA Soil DNA Kit (D5625-01) (Omega Bio-Tek, Norcross, GA, USA) was used to extract DNA from samples of the intestinal contents. The DNA was then frozen at -20°C for further analysis. The quantity and quality of DNA are confirmed using agarose gel electrophoresis and the NanoDrop NC2000 spectrophotometer (Thermo Scientific, MA, USA) respectively. The V3-V4 region of the 16S rRNA gene was amplified from the isolated DNA using the forward primer 338F (5’-ACTCCTACGGGAGGCAGCA-3’) and the reverse primer 806R (28). PCR amplification system (25 μL) consisted of 5×reaction buffer 5 μL, 5×GC buffer 5 μL, dNTP (2.5 mM) 2 μL, forward primer (10uM) 1 μL, reverse primer (10 uM) 1 μL, DNA template 2 μL, ddH2O 8.75 μL, Q5 DNA polymerase 0.25 μL. The following thermal cycle conditions were adopted: initial denaturation at 98°C for 2 min, denaturation at 98°C for 15 s, annealing at 55°C for 30 s, extension at 72°C for 30 s, and final extension at 72°C for 5 min.

The quantification of PCR amplicons was performed utilizing the Quant-iT PicoGreen dsDNA Assay Kit (Invitrogen, Carlsbad, CA, USA), after purification with Vazyme VAHTSTM DNA Clean Beads (Vazyme, Nanjing, China). Following individual quantification, the amplicons were combined in equal proportions, and pair-end 2×250 bp sequencing was conducted using the Illlumina NovaSeq (Illumina, San Diego, CA, USA) platform with the NovaSeq 6000 SP Reagent Kit (28, 29). All raw sequences were deposited in the NCBI Sequence Read Archive under accession number: PRJNA1119456 (https://www.ncbi.nlm.nih.gov/sra/PRJNA1119456).

#### 2.2.2 Bioinformatics and statistical analysis

Microbiome bioinformatics were performed with Quantitative Insights into Microbial Ecology II (QIIME2) (30). DADA2 (31) was used to filtered, denoised, merged and removed chimera from the data. To obtain amplicon sequence variants (ASVs), sequences were aligned to the SILVA Release 132 (http://www.arb-silva.de/) database and classified at the different classification levels with feature-classifier in QIIME2 (32). Alpha diversity metrics were calculated using Observed species index, Shannon diversity index (33), and Faith’s Phylogenetic Diversity (PD) (34). Beta diversity analysis was performed to investigate the structural variation of microbial communities across samples using Bray-Curtis matrix (35) and visualized via principal coordinate analysis (PCoA). We present the composition of ASV for each sample and groups at the phylum and genus levels in bar charts. LEfSe (Linear discriminant analysis effect size) (36) was performed to detect differentially abundant taxa across groups and identified biomarkers that differ significantly between groups. Taxa that achieved an Linear Discriminant Analysis (LDA) score>=4 and *P*<0.05 were identified as biomarkers.

### 2.3 Small intestine mucus metabolomic analysis

Metabolic profiles of PPs in the duodenum, jejunum, and ileum of Bactrian camels were compared using a non-targeted LC-MS/MS approach to analyze metabolite alterations. Eighteen samples and six Quality control (QC) samples were tested in POS and NEG modes using LC-MS/MS. For each cotton swab sample, add 400 μL of the extract solution (methanol: acetonitrile: water = 2: 2: 1). After 4 min of homogenization at 35 Hz, it was sonicated for 5 min in ice-water bath. The mixture was centrifuged for 15 min at 12000 rpm at 4°C after being precipitated at -40°C for 1 h. All samples were transferred to the Eppendorf tubes with an equal amount of 700 μL supernatant, and the supernatant was vacuum-concentrator dried. Add 220 μL extract (acetonitrile: methanol: water = 2: 2: 1, containing isotopically labeled internal standard mixture) to redissolve, centrifugation, and ultrasonic. The supernatant was collected into a new glass vial for LC-MS/MS analysis. To create QC samples, the same volume of supernatant was combined with each sample (37, 38).

LC-MS/MS analysis used Ultra High-Performance Liquid Chromatography system (UHPLC) (Vanquish, Thermo Fisher Scientific). Including a UPLC BEH Amide column (2.1 mm × 100 mm, 1.7 μm), and a Q Exactive HFX mass spectrometer (Orbitrap MS, Thermo). The target compounds were chromatographically separated using liquid chromatographic columns. The mobile phase consisted of 25 mmol/L ammonium acetate and 25 ammonia hydroxides in water (pH = 9.75) (A) and acetonitrile (B). The auto-sampler temperature was 4°C, and the injection volume was 2 μL. QC samples were added to the testing process to monitor the stability of the instrument. The acquisition software (Xcalibur v4.1, Thermo) regulated the mass spectrometer in order to acquire the complete scan MS spectrum. For the ESI source, the capillary temperature was set to 350°C, the sheath gas flow rate was 30 Arb, and the auxiliary gas flow rate was 25Arb. The full MS resolution was set to 120,000, the MS/MS resolution was set to 7500, the collision energy was 10/30/60 in NCE mode, and the spray voltage was 3.6 kV (positive ion modes) or -3.2 kV (negative ion modes). POS mode and NEG mode detection are both utilized to expand the detection area of substances.

ProteoWizard was used to convert the original data into mzXML format, and the R package, XCMS (39) was used to perform peak detection, extraction, alignment, and integration. The metabolites were annotated using the Kyoto Encyclopedia of Genes and Genomes (KEGG) and the Human Metabolome Database (HMDB) to confirm the specific taxonomic information of the metabolites. Principal component analysis (PCA) utilizing multivariate statistical analysis. The results were filtered based on variable importance in projection (VIP)>1.5, *P*<0.05, and fold change (FC) >= 2 or <=0.5 to identify statistically significant targets. The levels of different metabolites in each comparison were visualized in heatmaps. The differential metabolites were collected using MetaboAnalyst v5.0 (https://www.metaboanalyst.ca/), followed by Pathway Analysis (integrating pathway enrichment analysis and pathway topology analysis) and visualization. To investigate the correlation between differential metabolites and intestinal bacteria in Bactrian camels, a correlation analysis was performed using Pearson’s correlation coefficients. A comparison was conducted between the three regions using pairwise comparisons. All untargeted metabolomic data used in this study have been deposited to the EMBL-EBI MetaboLights database with the identifier MTBLS10376. The complete data set can be accessed at https://www.ebi.ac.uk/metabolights/MTBLS10376.

### 2.4 Determination of IgA, IgG and IgM expression levels

Tissue D-PPs J-PPs and I-PPs were weighed for 0.5g, and three biological replicates were generated for each group. Add 1mL of PBS and two magnetic beads, homogenize for 15 min at -10°C before centrifuging for 10 min at 4°C at 12,000 rpm (22). Retrieve the supernatant. The protein content was measured using the BCA protein Assay Kit (Solarbio, Beijing, China). The expression levels of IgA, IgG and IgM were assessed in various groups using ELISA kits (IgA ELISA Kit, YJ962011, Shanghai Enzyme Linked Biotechnology, China; IgG ELISA Kit, YJ962012, Shanghai Enzyme Linked Biotechnology, China; IgM ELISA Kit, YJ962013, Shanghai Enzyme Linked Biotechnology, China). One-way analysis of variance (ANOVA) was performed using SPSS v23.0 (SPSS Inc., Chicago, USA) examine the differences between the three groups and was used for statistics. All experimental results were represented as mean ± standard deviation (SD).

## 3 Results

### 3.1 Microbiota analysis in PPs of small intestine

#### 3.1.1 Microbiome analysis and diversity analysis

A total of 920409 sequences were acquired from the 16S rRNA sequencing for 18 intestinal samples using the Illlumina NovaSeq-PE250 platform; on average, 51133 sequences were obtained per sample. 11063 ASVs were obtained in total following sequence clustering (**Supplementary Table S1**). On average the most ASVs were identified from D-PPs (5941 ASVs), comparing to J-PPs of 4188 ASVs and I-PPs of 3441 ASVs. The rarefaction curve shows all samples have reached sufficient sequencing depths for analysis as the numbers of ASVs have reached plateaus (**Supplementary Figure S1**).

**Figure 1:**
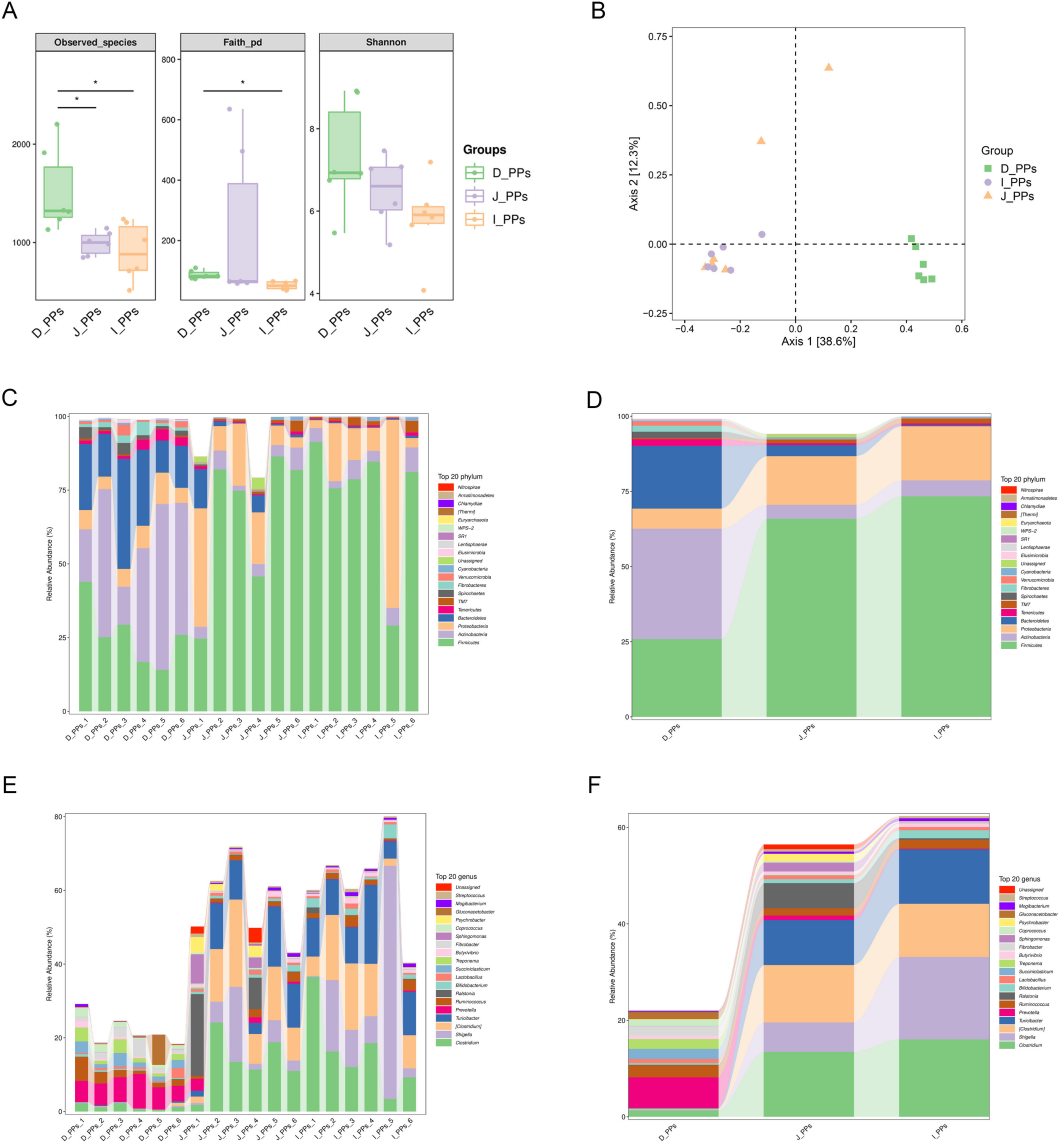
Results of microbiome diversity and composition analysis. **(A)** Differences in Alpha diversity between D-PPs, J-PPs and I-PPs, * P-value <0.05. **(B)** Differences in Beta diversity among the three groups were analyzed by PCoA based on the Bray-Curtis distance matrix. Relative abundance of the top 20 taxon of samples within each group **(C)** and between groups **(D)** at the phylum level. Relative abundance of the top 20 taxon of samples within each group **(E)** and between groups **(F)** at the genus level.

To achieve a comprehensive assessment of the microbial community as a whole, the within-habitat diversity and between-habitat diversity of species were evaluated using Alpha and Beta diversity (40), respectively. An analysis of alpha diversity revealed a statistically significant difference in the observed species index between the D-PPs and J-PPs groups and the D-PPs and I-PPs group (*P < 0.05*). Faith’s PD identified statistically significant distinctions between I-PPs and D-PPs (*P<0.05*). However, the Shannon index, a measure of species richness, showed no significant differences among the three groups (**Figure 1A**). The Bray Curtis-based PCoA analysis showed that samples from the D-PPs group formed a distinct cluster separate from the other two groups, while samples from the J-PPs group and I-PPs group clustered together (**Figure 1B**). The connection between the jejunum and ileum suggests that their microbiota varieties are similar.

#### 3.1.2 Microbiological characteristics of different intestinal segments

A grand total of 29 taxonomies were ascertained at the phylum level. There are 25 phyla in the D-PPs group, 24 in the J-PPs group, and 21 in the I-PPs group. The top 20 relative abundant phyla were determined for each type of the tissue (**Figures 1C, D**). Despite the dissimilar abundances, *Firmicutes*, *Proteobacteria*, and *Actinobacteria* are the predominant bacteria found across all three groups. The mean abundances of *Firmicutes* are 65.9% and 73.4% in J-PPs and I-PPs, respectively. The mean abundances of *Proteobacteria* are 16.9% and 18% in J-PPs and I-PPs respectively. D-PPs has the lowest abundance of *Firmicutes* for 25.9% and *Proteobacteria* for 6.7% among the tissues. *Actinobacteria* is the most abundant phylum in D-PPs with 20.8%, compared to J-PPs (4.6%) and I-PPs (5.3%). *Bacteroidetes* exhibited a gradual decrease in the mean abundance of D-PPs, J-PPs, and I-PPs, reaching levels of 36.7%, 3.7%, and 0.4%, respectively. **Supplementary Figure S2A** provides the results of the difference analysis for the top 20 taxonomies at the phylum level.

A total of 357 taxonomies were identified at the genus level, with 252 in D-PPs, 271 in J-PPs, and 208 in I-PPs for each group. **Figures 1E, F** displays the top 20 genera based on the mean abundance of samples from each group. The J-PPs and I-PPs are mostly composed of the primary genus *Clostridium* and *Turicibacter*. The mean abundance ranges from 9% to 16%. In D-PPs, *Turicibacter* has an abundance of less than 1%, except for *Clostridium*, which has an abundance of 1.3%. *Prevotell* is the most prevalent genus in D-PPs, with an average abundance of 6.3%. Conversely, the mean abundance of *Prevotella* in J-PPs and I-PPs is below 1%. *Shigella* was most abundant in the I-PPs group, with a prevalence of 17%, compared to 6.1% in the J-PPs and 0.2% in the D-PPs. **Supplementary Figure S2B** provides the results of the difference analysis for the top 20 taxonomies at the genus level. In addition, analysis of the top 20 taxonomies down to the species level indicated five species with complete information (**Supplementary Figure S3**).

#### 3.1.3 Microbiota biomarkers in different intestinal segments

**Figure 2:**
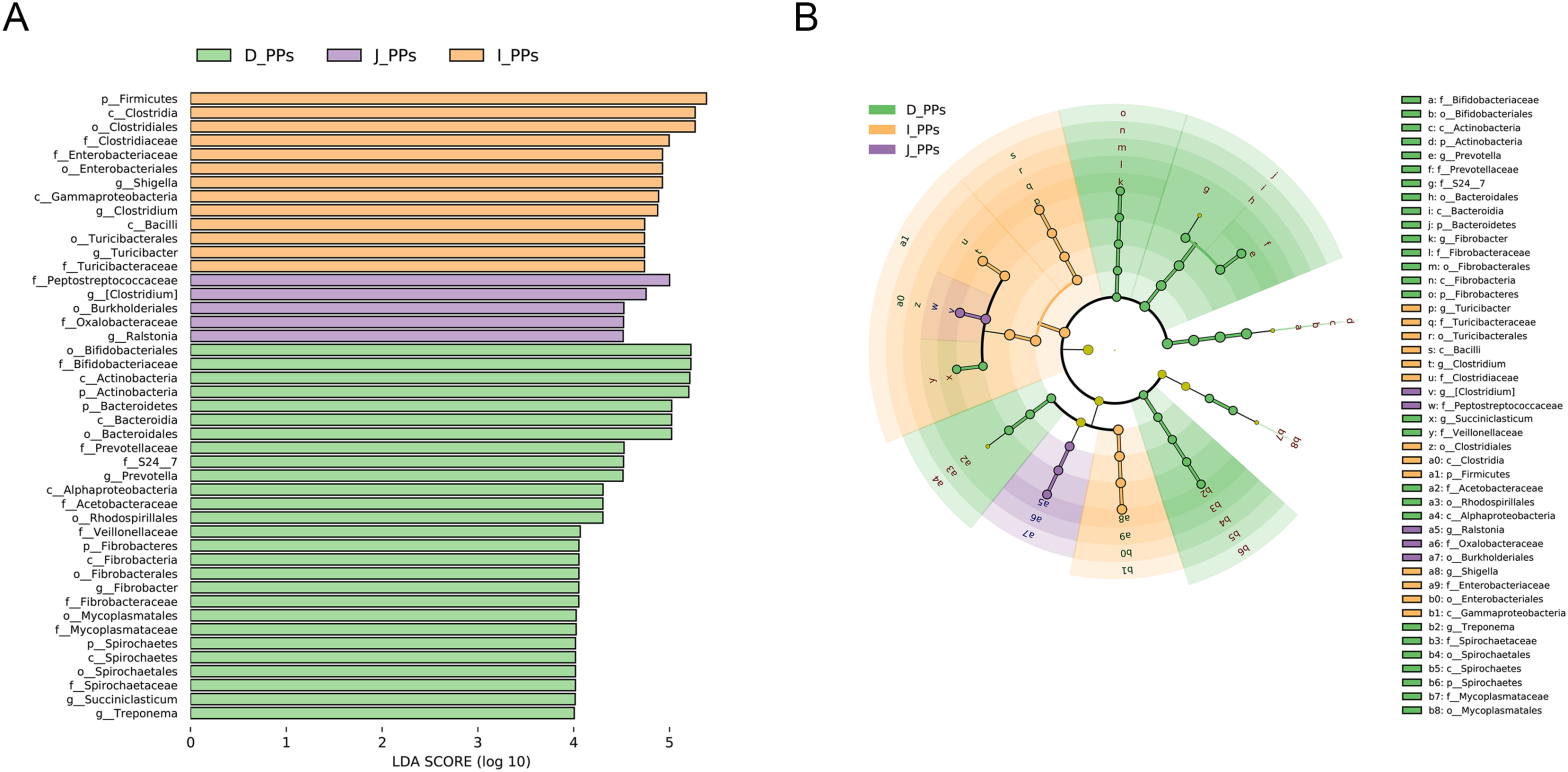
LEfSe analysis results. **(A)** Histogram of the LDA scores (LDA>4). **(B)** Cladogram of microbiota in different groups.

The histogram of the LDA value distribution indicates 45 taxa with significant differences across various taxonomic levels (*P*<0.05) (**Figure 2A**). The main contributing bacteria varied throughout several regions of the small intestine. Genera *Prevotella*, *Fibrobacter*, *Succiniclasticum*, and *Treponema* are significantly enriched in D-PPs. Likewise, at the genus level, *Ralstonia*, *Shigella*, *Clostridium*, and *Turicibacte*r in J-PPs and I-PPs was significantly higher than D-PPs. **Figure 2B** shows that several genera of *Firmicutes* have been discovered in all three groups on the cladogram, including *Succiniclasticum* (D-PPs), and *Turicibacter*, *Clostridium* (I-PPs, J-PPs). Within the phylum *Fibrobacteres*, *Spirochaetes*, and *Bacteroidetes*, only bacteria taxonomically classified as belonging to the D-PPs were found.

### 3.2 Metabolic analysis in PPs of small intestine

**Figure 3:**
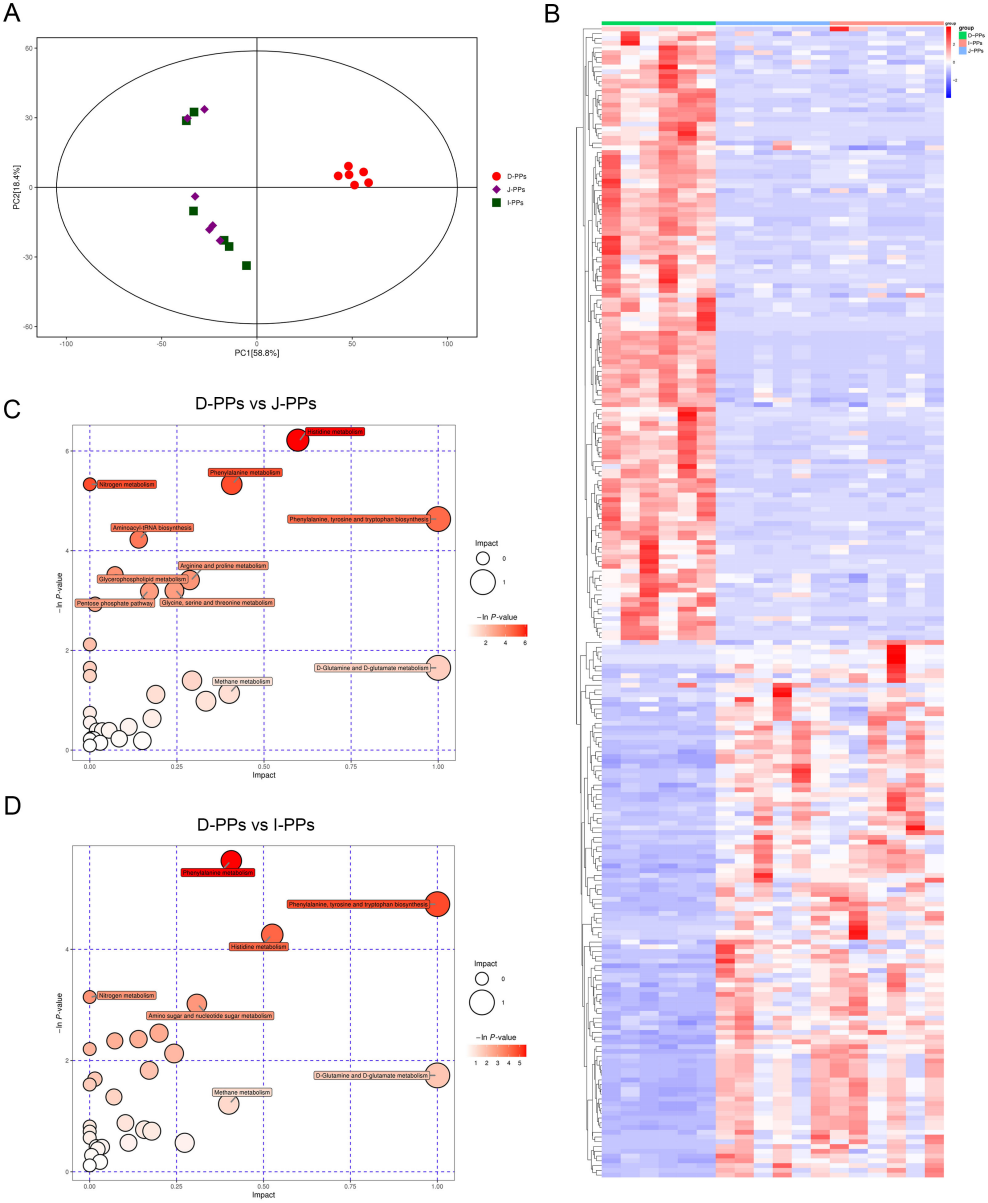
Differences in metabolites between D-PPs, J-PPs and I-PPs. **(A)** Score plots of the PCA models. **(B)** Heatmap of differential metabolites between groups (VIP > 1.5, *P* < 0.05). Pathway Analysis of differential metabolites between group D-PPs and J-PPs **(C)**, group D-PPs and I-PPs **(D)**. The ordinate and bubble color are P-values (natural negative logarithms) in enrichment analysis. The horizontal coordinate and bubble size represent the impact factor of the pathway in topological analysis.

Monitor the instrument stability and signal in real time to ensure the quality of the final data acquisition. The Extracted Ion Chromatogram (EIC) of QC samples showed that the peak retention times of all samples overlapped with the chromatographic peaks of total ions, indicating high stability in instrument data acquisition (**Supplementary Figure S4A**). Additionally, the correlation of all QC samples was above 0.9, demonstrating the high reliability of the experimental data (**Supplementary Figure S4B**). A total of 1006 metabolites were identified in all intestinal samples under POS and NEG modes (**Supplementary Table S2**). As indicated, J-PPs and I-PPs were not effectively isolated (**Figure 3A**). Samples from D-PPs were clustered and distanced with the remaining two groups This feature is consistent with the results of the above intestinal microbiological analysis. All differential metabolites were visualized using a cluster heat map (**Figure 3B**). A total of 191 metabolites exhibited differential expression between D-PPs and J-PPs (107 up-regulated, 84 down-regulated), and 194 metabolites were significantly differential expressed between D-PPs and I-PPs (108 up-regulated, 86 down-regulated), as determined by VIP > 1.5, *P* < 0.05; FC>=2 or FC<=0.5. Compare to other comparisons, less significantly differential metabolites identified between the J-PPs and I-PPs, six distinct metabolites exhibited differed (two down-regulated and four up-regulated). The specific information about the difference metabolites can be obtained from **Supplementary Table S3**.

The most abundant metabolites found in J-PPs and I-PPs include carboxylic acids and their derivatives, such as L-Glutamic acid, L-Histidine, L-Tyrosine, and L-Arginine; organonitrogen compounds, such as choline and N-Lactoyl ethanolamine; fatty acyls, such as 3-hydroxyvalproic acid and 2-ethylacrylic acid; and organooxygen compounds, such as 4-Hydroxybenzaldehyde.In contrast, the D-PPs groups exhibited higher levels of carboxylic acids, and derivatives (Threoninyl-Leucine, Aspartyl-Glutamate, Valyl-Proline, Phenylalanyl-Arginine); fatty Acyls (Propionylcarnitine, Adipic acid); organooxygen compounds (Glucose 6-phosphate); and organonitrogen compounds (Acetylcholine, Histamine). The D-PPs exhibited a greater variety and abundance of compounds that contribute to glucose and lipid metabolism in comparison to the other two groups. These compounds consisted of D-Ribulose 5-phosphate, Glucose 6-phosphate, Pantothenol, Adipic Acid, Sebacic Acid, and Acylcarnitine, just to name a few. Metabolites of the three groups consisted primarily of carboxylic acids and their derivatives, although the specific types varied. D-PPs contain primarily polypeptide molecules, whereas J-PPs and I-PPs are abundant in free amino acids. It is suspected that this is connected to the specific positioning of PPs in the duodenum of the Bactrian camel and the digestive processes of the small intestine.

Subsequently, Pathway Analysis of differentiated metabolites between groups was used to enhance comprehension of the biological activities of certain metabolites in different regions of the small intestine. Thirty-four pathways were annotated in D-PPs and J-PPs, with nine significantly enriched (*P*<0.05). Additionally, 31 pathways were annotated in D-PPs and I-PPs, with five significantly enriched pathways (*P*<0.05). The outcome of the pathway between J-PPs and I-PPs is ambiguous. Pathways with statistical significance (*P*<0.05) and the impact of 1 were illustrated by the bubble map. Such as histidine metabolism, phenylalanine metabolism, arginine and proline metabolism, glycerophospholipid metabolism, and pentose phosphate pathway (**Figures 3C, D**; **Supplementary Table S4**). To summarize, the metabolic differences between D-PPs, J-PPs, and I-PPs primarily revolve around the metabolic and biosynthesis for various amino acids, along with certain processes related to lipid and glucose metabolism.

### 3.3 Cross-correlation analysis between the microbiota and metabolites

**Figure 4:**
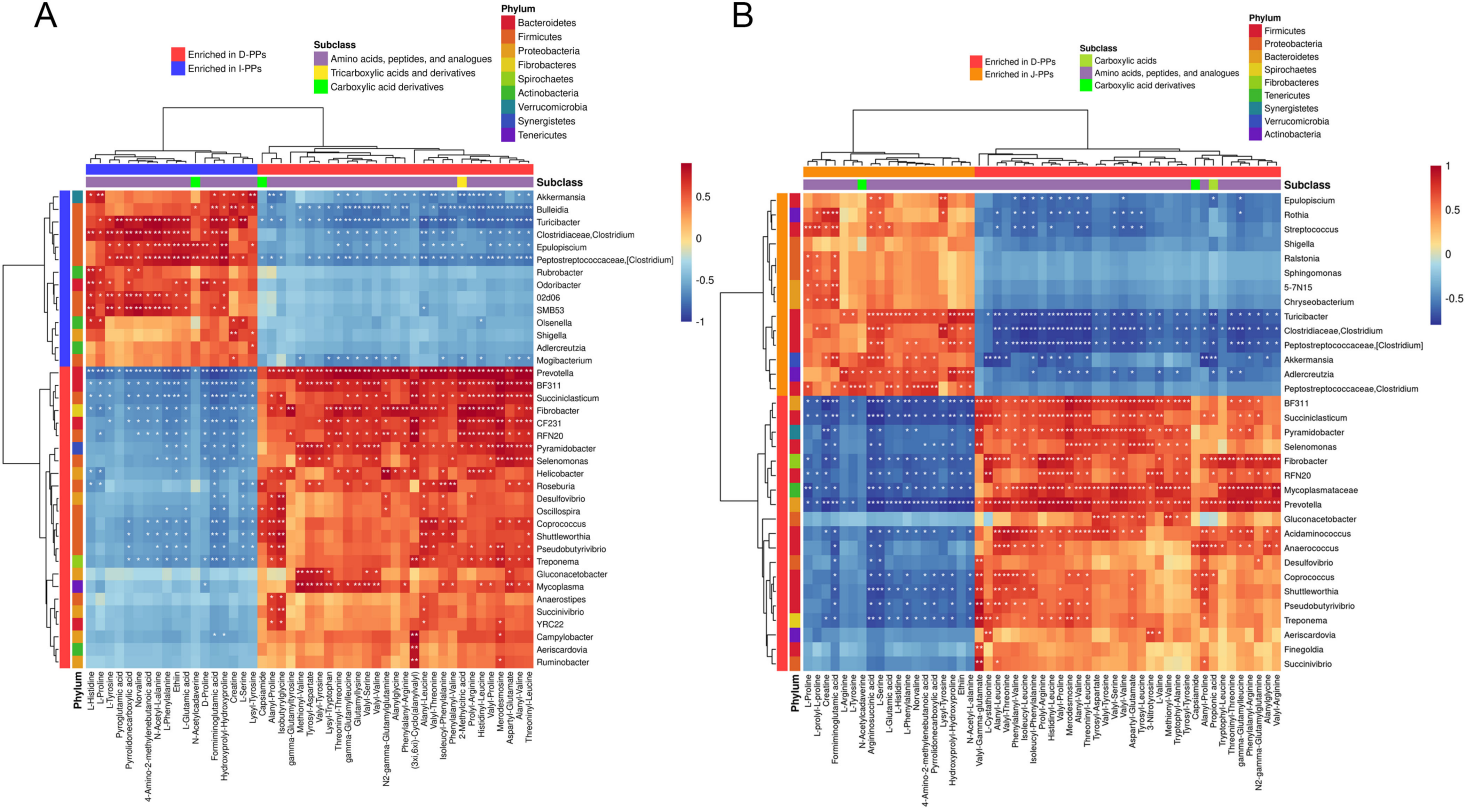
Cross-correlation analysis between microbiota and metabolites. Heatmap of the correlation between microbiota with statistical differences at genus level and the differential metabolites of carboxylic acids and derivatives. **(A)** Group D-PPs and I-PPs. **(B)** Group D-PPs and J-PPs. The horizontal coordinate shows the differentially abundant metabolites and their subclasses, while the vertical coordinate shows the differentially abundant bacteria and their phylum classification. Red represents positive correlation, blue represents negative correlation, and white stars represent significant correlation areas (* *P* < 0.05; ** *P* < 0.01; *** *P*< 0.001).

The differential metabolites between the groups (D-PPs vs J-PPs: 161; D-PPs vs I-PPs: 161) and ASVs with significant differences (*P* < 0.05) at the genus level (D-PPs vs J-PPs: 33; D-PPs vs I-PPs: 38) were selected for correlation analysis (**Supplementary Figure S5**). While, correlation analysis was also performed on the differential metabolites belong to carboxylic acids and derivatives, and differential bacteria considering the mentioned findings (**Figures 4A, B**). *Prevotella* was negatively correlated with metabolites of J-PPs and I-PPs (*P* < 0.01), but positively correlated with all amino acids, peptides, and analogues in D-PPs (*P* < 0.01). *Fibrobacter*, S*ucciniclasticum*, and *BF311* exhibit comparable properties. Significant positive correlations (*P* < 0.05) were observed between *Turicibacter*, *Akkermansia*, and *Clostridium* (*Clostridiaceae*) with most amino acid metabolites in J-PPs and I-PPs. Conversely, a negative correlation (*P* < 0.05) was observed between metabolites and bacteria in D-PPs. The correlation analysis revealed that the most enriched bacteria in the three groups’ samples had a strong correlation with their amino acid metabolites.

### 3.4 Expression of IgA, IgG, and IgM in different segments of the small intestine

**Figure 5:**
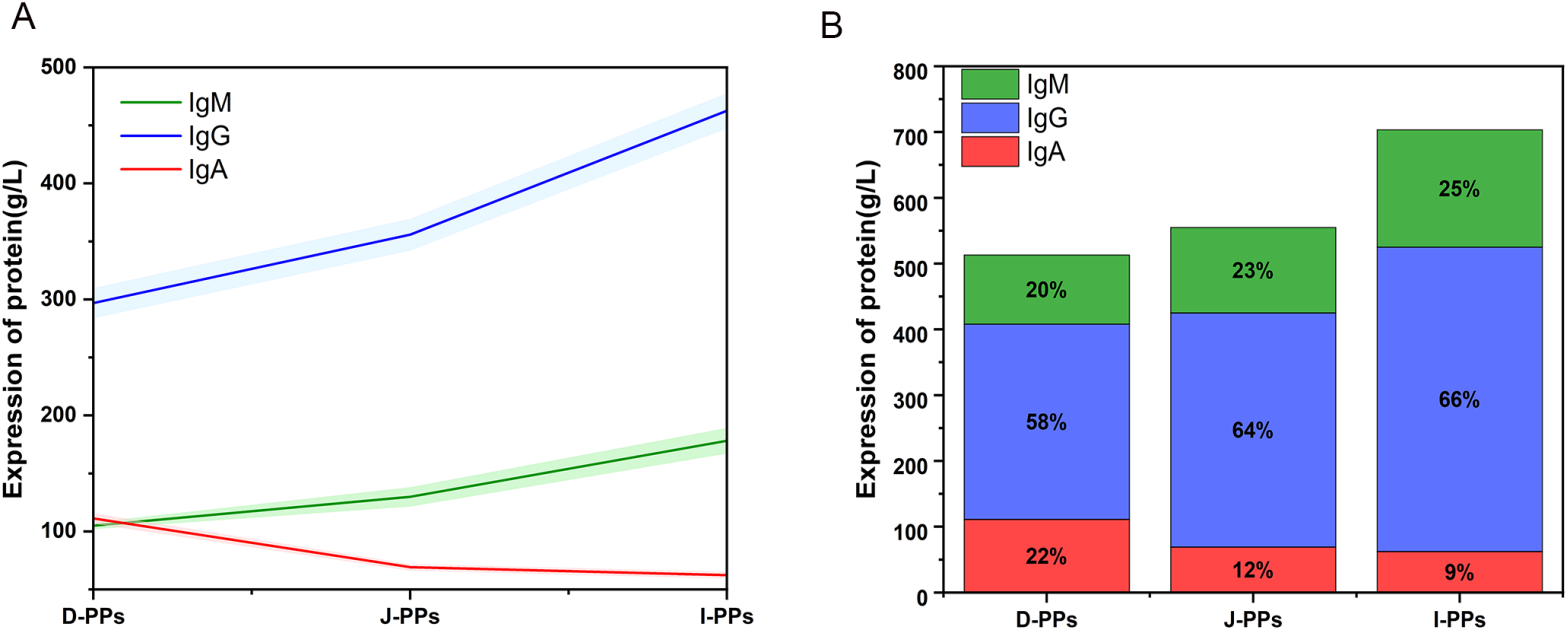
Results of IgA, IgG, and IgM in D-PPs, J-PPs, and I-PPs groups with ELISA. **(A)** A line chart displays the patterns of IgA, IgG, and IgM expression trends across the three groups. The results are presented as mean ± SD (the shaded regions represent the range of the SD). **(B)** The distribution of IgA, IgG, and IgM expression in the three groups is represented by a stacked bar chart.

ELISA results showed that I-PPs had considerably higher levels of IgG and IgM compared to J-PPs and D-PPs (*P* < 0.01) (**Figure 5**). D-PPs had the greatest expression level of IgA, which has statistical difference from J-PPs and I-PPs (*P* < 0.01) (**Table 1**). Overall, the proportions of immunoglobulins were comparable in both I-PPs and J-PPs, but the level of IgA in D-PPs was distinct, setting it apart from the other two groups.

**Table 1:** Results of differential analysis of IgA, IgG, and IgM expression levels in D-PPs, J-PPs, and I-PPs.

Comparisons	P-vaule		
	IgA	IgG	IgM
D-PPs vs. J-PPs	6.030E-06	2.061E-03	9.528E-03
D-PPs vs. I-PPs	2.513E-06	6.591E-06	3.340E-05
J-PPs vs. I-PPs	5.532E-02	8.327E-05	3.471E-04

## 4 Discussion

This work enhances our understanding of the distinct characteristics of metabolites and the microbial composition in several immune induction sites of the small intestine in Bactrian camels and examines their interrelationships. The results showed that D-PPs were significantly different from J-PPs and I-PPs in terms of both the commensal microbiota and their metabolites in the digestive tract. There was no statistically significant distinction observed between J-PPs and I-PPs.

Gut microbes play a vital role in nutrient absorption and maintaining immune homeostasis, with variations in microbial communities between intestinal segments being indicative of the distinct digestive and immune requirements of the gut. In the diversity analysis of Bactrian camel intestinal flora, there was no significant difference in the Shannon diversity index between the duodenum and ileum, but a significant difference in Faith’s PD. The duodenum showed greater phylogenetic diversity than the ileum, indicating that the bacterial species in these two regions are more diverse and distinct in their evolutionary relationships. The intestinal microbiota of Bactrian camels primarily consists of *Proteobacteria*, *Actinobacteria*, and *Firmicutes*, with distinct distribution patterns compared to other ruminants. Bactrian camels have a higher abundance of *Actinobacteria* and a lower proportion of *Bacteroidetes* in their small intestines than cattle (41)and sheep (42). Uniquely, the distribution characteristics of *Firmicutes* are different from those of other ruminants but consistent with the research results of the intestinal mucous microbiota in mice (43). Except the species, the composition of the intestinal microbiota is also related to diet, habitat, and environmental circumstances in captivity or the wild. Bactrian camels, adapted to alternating between grazing and captivity, primarily consume *Nitraria tangutorum*, *Agriophyllum pungens*, and *Ceratoides latens (* 23). This management model contributes to the unique characteristics of their intestinal flora. *Prevotella*, *Fibrobacter*, and *Succiniclasticum* are the dominant genera in the D-PPs, with *Prevotella* being the most abundant. As a member of the *Bacteroidetes*, *Prevotella* plays a key role in the breakdown of polysaccharides and is often considered a beneficial bacterium in plant-based diets. To be more specific, *Prevotella* colonization is linked to an increased abundance of genes encoding glycoside hydrolases and cellulases in the gut (44). Additionally, both *Prevotella* and *Succiniclasticum* have been shown to significantly promote glucose metabolism and improve glucose tolerance (44, 45). This aligns with the significant enrichment of glucose metabolism-related compounds, such as glucose 6-phosphate, D-ribulose 5-phosphate, and gluconolactone, in the D-PPs. *Fibrobacter*, another genus involved in the breakdown of xylan and cellulose (46), also exhibited higher abundance in the D-PPs. These findings explained the greater capacity for carbohydrate and cellulose catabolism that the duodenum has for the Bactrian camel compared to other intestinal regions. In the gut, *Succiniclasticum* plays a big role in making short-chain fatty acids (SCFAs) by turning succinic acid into propionic acid, which makes butyrate more bioavailable (47). In the J-PPs and I-PPs, *Clostridium* and *Turicibacter* are the dominant genera, both known for promoting gut health through the production of SCFAs such as butyrate and propionate (48, 49). Noticeably, while no significant differences were found in SCFA levels across different intestinal segments, there were notable differences in the enrichment of amino acids, which may serve as precursors.

The duodenum, serving as the route through which chyme is transferred from the stomach to the small intestine, is exposed to a high concentration of food antigens. It requires an abundance of commensal microbiota and active autonomic secretion activity to aid in nutrient digestion in the host. The *Prevotella* synthesizes sulfosulfonase, facilitating the entry of glycosidase to degrade host mucin (50). This function enhances intestinal permeability and facilitates the efficient exchange of substances. Bactrian camels have a high abundance of *Prevotella* in their duodenum, which creates favorable conditions for digestion and absorption. However, increased *Prevotella* abundance can result in intestinal metabolic abnormalities, worsen inflammatory reactions, and contribute to autoimmune diseases (51). The lipopolysaccharides produced by *Prevotella* can stimulate the expression of multiple pro-inflammatory cytokines, activate lysosomal enzyme production, and increase phagocyte proliferation (52). Compared to other parts of the intestine, the duodenum has a higher concentration of inflammatory cells, such as CD103^+^ CD11b^+^ dendritic cells and Th17 cells (9). This is most likely caused by the abundance of food antigens in the duodenum and the pro-inflammatory effects of *Prevotella* intestinal colonization. In contrast, in the J-PPs and I-PPs, the microbiota composition and the induced immune responses differ. *Clostridium*, for example, promote the accumulation of Treg cells, which drive the production of anti-inflammatory CD4^+^FoxP3^+^ Tregs and interleukin-10 (IL-10) in a transforming growth factor beta (TGF-β)-rich environment (53, 54). A new gut-derived Treg subpopulation, CCR6^+^ CXCR6^+^ DP8α Treg, responds to *Clostridium* and enhances IL-10 production through CD39 dependent mechanisms, reinforcing their anti-inflammatory role (55). *Clostridium* and its metabolites can also activate innate lymphoid cells and mucosal-associated invariant T cells, further driving immune responses (56). Additionally, *Turicibacter* can transform bile acids in the host gut and modulate immune responses through bile acid receptor expression in various immune cell types (57). The ileum serves as the primary location for bile acid absorption, aligning with the observation that *Turicibacter* is notably abundant in I-PP (58). Our prior research has indicated that the terminal section of the ileum in Bactrian camels has the most intense distribution of PPs in the small intestine (25). Therefore, the ileum is likely to exhibit the most active immune effect. The variation in lymphoid tissue distribution is a contributing factor to the differences in bacterial community structure (8). Overall, the D-PPs, J-PPs, and I-PPs in the small intestine of the Bactrian camel exhibit the characteristics of microbiome regionalization. We suggest that this arrangement is the outcome of a reciprocal selection process between the host and the intestinal microbiota.

Amino acids contribute to the development of intestinal immune cells, tissue homeostasis, and immune response mechanisms. Previous research suggests that amino acids are the primary metabolites found in the small intestine (17). Our research revealed that D-PPs have the highest concentration of polypeptides, including Prolyl-Arginine, Valyl-Proline, Valyl-Serine, and Isoleucyl-Phenylalanine. Whereas, the jejunum and ileum are plentiful in tiny molecular amino acids, including L-Arginine, L-Serine, L-Proline, L-Phenylalanine, and L-Glutamic acid. D-PPs has a distinct structure compared to the middle and posterior segments of the duodenum, which is in the duodenum’s oncoides, and attaching to the abomasum. It receives polypeptide molecules from the initial digestion in the abomasum, which are subsequently degraded by peptidase enzymes in the small intestine into free amino acids, or dipeptide and tripeptides for absorption (59). The primary source of absorption for most amino acids is the small intestine (60). However, the rate of absorption varies among different amino acids due to differences in the intestinal segments (59, 61). The difference in the arrangement of amino acid transporters in the intestinal segment is what determines this (62). Given these characteristics, we propose that the differential distribution of amino acid transporters in the small intestine of Bactrian camels may adapt to the nutrient absorption and immune function requirements of different intestinal segments. Arginine is considered a metabolic center that controls the immune response. Insufficient levels of arginine have a detrimental impact on both proliferation and activation of T cells, but can be remedied by taking arginine supplements (63). Changes in arginine levels can lead to differences in T cell development and activation between J-PPs and I-PPs with the D-PPs. In addition, arginine supplementation has been shown to decrease interleukin-17 levels, alleviate inflammation, and promote a reorganization of the intestinal microbiota in the direction of *Bifidobacterium* enrichment (64). Therefore, the discrepancy in immune cell distribution can be attributed to the variance in arginine concentration across the distinct segments of the small intestine. Adaptive immune responses may more prevalent in the middle and posterior regions of the small intestine compared to the duodenum, which has an elevated level of inflammatory response factors. L-Arginine serves as a precursor to many bioactive chemicals, including polyamines, proline, and creatine, helping in maintaining a healthy mucus barrier in the gut (65) and regulating the immunological balance of intestinal microbes (66, 67).The high expression of L-Arginine in the J-PPs and I-PPs of Bactrian camels may indicate evidence of intestinal segmentary immunity. Abundant amino acids in the middle and posterior segments of the small intestine support the regular activity of immune cells and influence gut microbiota-host crosstalk. Amino acids with similar functions, such as L-Proline (68), L-glutamic acid (69), Hydroxyproline (70) and L-Serine (71), are also enriched in the jejunum and ileum.

The differential metabolites with high expression in D-PPs help us better understand the regional traits of small intestine in camel. For example, the contents of acetylcholine, histamine, and serotonin were particularly significant in the duodenum, followed by the jejunum, and finally, the contents of PPs in the ileum. They can increase intestinal capillary permeability, regulate intestinal smooth muscle movement, participate in inflammatory responses, and change intestinal flora composition (72–75). Histamine enrichment suggests that some immune cells with histamine receptors, such as mast cells, basophil cells, and dendritic cells, are more active in the duodenum. Serotonin and histamine enhance intestinal permeability, facilitate the molecular transfer of nutrients, and build the daily immunological function of the intestine. As previously mentioned, the microbiota and high concentrations of food antigens present in the camel duodenum can induce inflammatory responses, further reinforced by the enrichment of histamine and serotonin. The varying levels of these chemicals in Bactrian camels demonstrate how the immune systems in the duodenum, jejunum, and ileum are regulated by different metabolites. Additionally, the analysis of IgA, IgG, and IgM expression revealed that the amount of IgA in D-PPs was significantly higher than in J-PPs and I-PPs. Studies have shown that the neutralization effect of IgA varies among different enterotypes. In intestinal environments dominated by *Prevotella*, *Bacteroides*, and *Clostridium*, IgA binding is significantly higher for *Prevotella* and *Bacteroides* than for *Clostridium (* 76). Our study aligns with these findings. In Bactrian camels, the duodenum, dominated by *Prevotella* and *Bacteroidetes*, showed a positive correlation with high IgA concentrations, while the jejunum and ileum, dominated by *Clostridium*, exhibited lower IgA levels. These results provide a clearer understanding of the unique immune functions of the Bactrian camel’s duodenal environment. IgA attaches to the mucus layer that covers the cells lining the intestines, preventing harmful germs from sticking to them and neutralizing antigen without triggering inflammation (77). The variation in immunoglobulin expression suggests that the body adjusts the immunological tactics of different sections of the gut to enhance defensive effectiveness and reduce tissue harm.

The composition of microbiota in the small intestine of the Bactrian camel changes across different regions. However, the contribution of variation in location to intestinal metabolism is unclear. The correlation analysis revealed varying degrees of correlation between the dominant genus in each segment and the enriched metabolites. Although a precise causal connection cannot be determined from this investigation, there are certain connections between the two that help in understanding the peculiarities of the ecosystem within the camel gut. *Prevtella* and *Succiniclasticum* in the duodenum of the Bactrian camel highly correlated with differential metabolites, such as histamine, serotonin, and polypeptide molecules. *Clostridium* and *Turicibacter* in jejunum and ileum were also significantly positively correlated with L-Glutamic acid, L-Proline, and L-Tyrosine. Many intestinal diseases are associated with intestinal microbiota disorders and metabolic abnormalities. Supplementation of some amino acids or probiotics in livestock diets can improve disease prevention, feed efficiency, and growth performance in animals. For example, *Prevtella* and *Clostridium* have been demonstrated to play an essential role in preventing diarrhea (78), reducing inflammation and allergic disorders (55), stimulating IgA production (78), enhancing animal growth performance and meat quality (79–81), and improving intestinal health.

Compared to other lymph nodes, the ratio of B cells to T cells in PPs is five times greater (82). This characteristic empowers PPs to produce a substantial quantity of antibodies, thereby serving as the secondary barrier of the intestinal immune system (83). IgA, IgG, and IgM are major antibody members. Like the results of the microbiome and metabolome, the D-PPs exhibited slightly different antibody levels compared to the J-PPs and I-PPs. Immunoglobulin levels were lowest in D-PPs, but IgA levels were significantly higher than in other sites. This is related to the special microenvironment of D-PPs of Bactrian camel. The distribution proportion of immunoglobulins in J-PPs and I-PPs groups was similar. However, the I-PPs had the highest content of immunoglobulins in the small intestine, corresponding to the highest distribution density of PPs in the ileum of Bactrian camels (25). The ileum, which is also the primary site for antigen sampling and adaptive immune induction. Study have shown that the mucous layer at the end of the ileum is thin and rich in IgM antibody-secreting cell (23). Bactrian camel ileum PPs accumulate in the terminal ileum, where the mucus is thin. The elevated degree of antibody expression observed in I-PPs may supplement the deficiencies of the mucus barrier. Furthermore, a decrease in mucus layer thickness facilitates antigen absorption, thereby initiating an immune response to eliminate the antigen (84). Based on our studies, we propose that the variation in IgA, IgG, and IgM levels in the small intestine of Bactrian camels is associated with the spatial distribution of microbiota and metabolites. Additionally, despite the variation in their total immunoglobulin content, the metabolites and microbiota of the J-PPs and I-PPs are not significantly different. Hence, additional research is necessary to clarify the underlying mechanisms of this phenomenon.

## 5 Conclusion

This study comprehensively summarizes the microbiota and metabolite characteristics of PPs in different regions of the Bactrian camel’s small intestine, as well as the differential expression of immunoglobulins. The findings indicated that the microbiota and metabolites of D-PPs exhibited unique characteristics compared to those of J-PPs and I-PPs. However, J-PPs and I-PPs displayed similarities in intestinal environment. Similar conclusions were drawn for immunoglobulin expression. This study provides important insights into the regionalization of intestinal immunity in unique animals. It offers a new perspective for disease prevention and breed management of Bactrian camels, enhancing our understanding of the immune and digestive characteristics of their intestinal mucosa.

## Data Availability

The datasets presented in this study can be found in online repositories. The names of the repository/repositories and accession number(s) can be found below: PRJNA1119456 (SRA), MTBLS10376 (Metabolights).
