# Supplementary material for: Regionalization of intestinal microbiota and metabolites in the small intestine of the Bactrian camel
Source: Front Immunol. 2024 Nov 26;15:1464664. doi: 10.3389/fimmu.2024.1464664 (PMC11628504; doi:10.3389/fimmu.2024.1464664)
Supplement: Supplementary file 1 [file DataSheet1.docx]

Supplementary Material

# Supplementary Figures


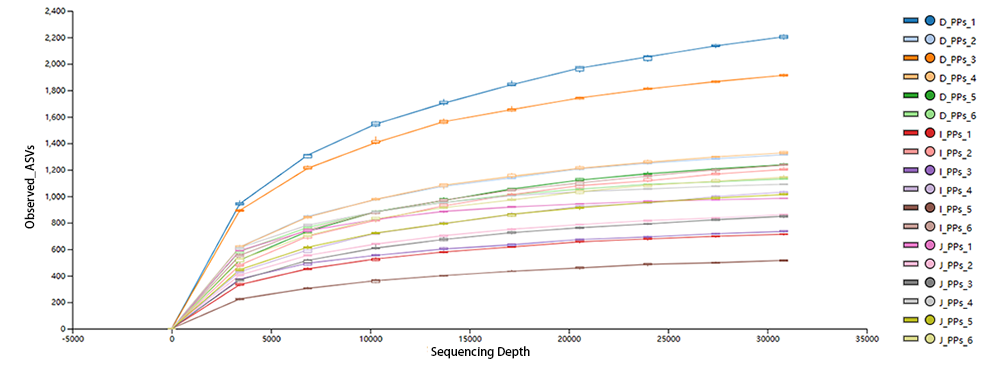


**Figure S1 The rarefaction curve of all samples.** The rarefaction curve shows all samples have reached sufficient sequencing depths for analysis as the numbers of ASVs have reached plateaus.


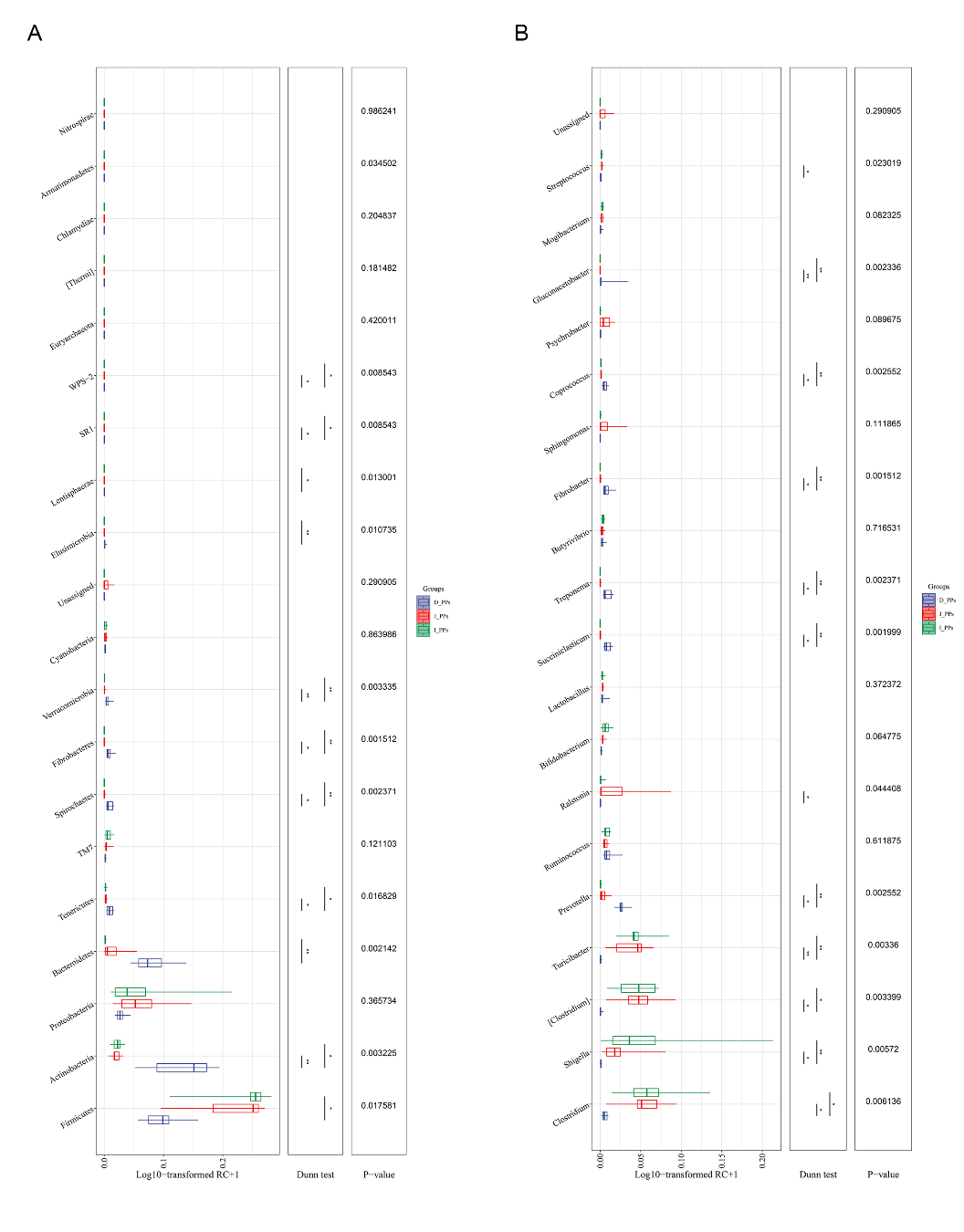


**Figure S2 Analysis of Top 20** **taxonomies in different groups at phylum and genus level. (A)**: Differential taxonomies in phylum level. **(B)**: Differential taxonomies in genus level. The three parts of the figure represent the following: the first displays the logarithmic transformation of abundance values; the second shows the results of the Dunn's test for intergroup comparisons, * *P* < 0.05; ** *P* < 0.01; and the third depicts the P-values obtained from the Kruskal-Wallis test across the three sample groups.


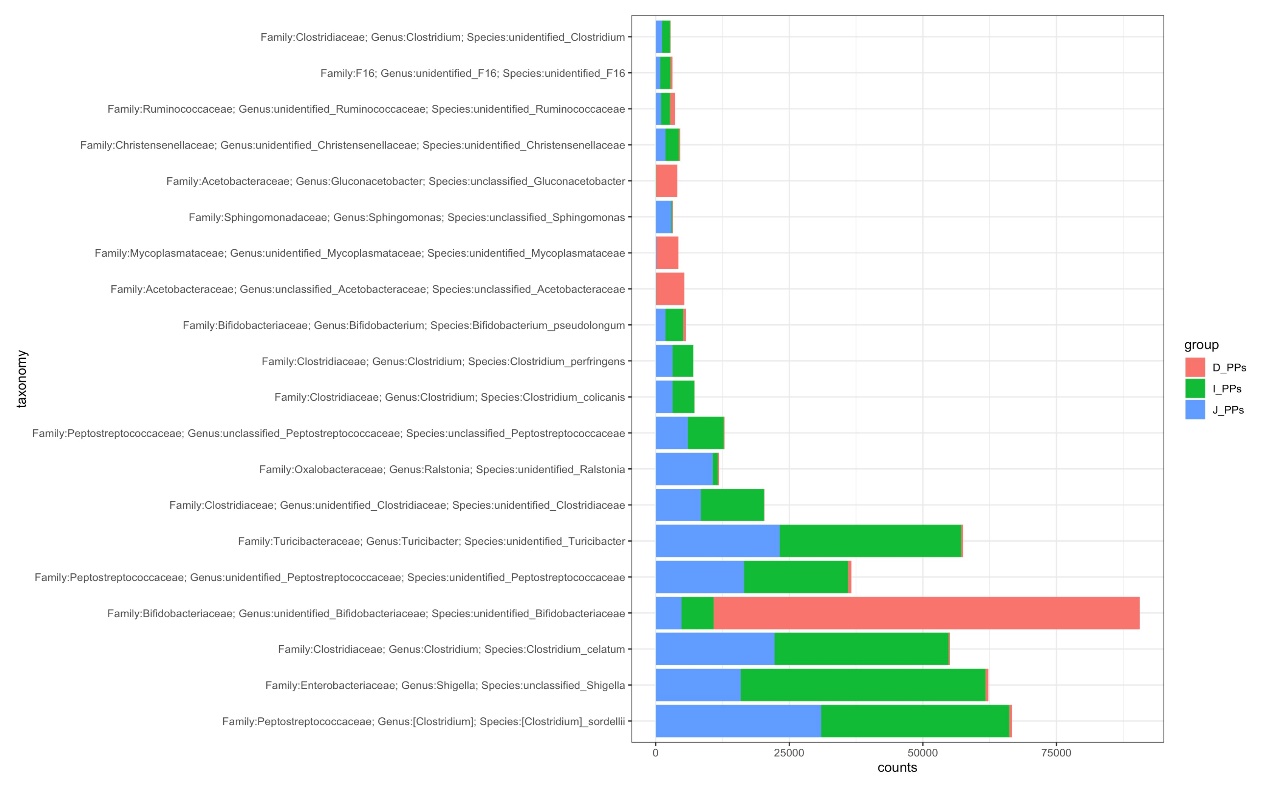


**Figure S3 Top 20 taxonomies with relative abundances of D-PPs, J-PPs, and I-PPs at species level.**


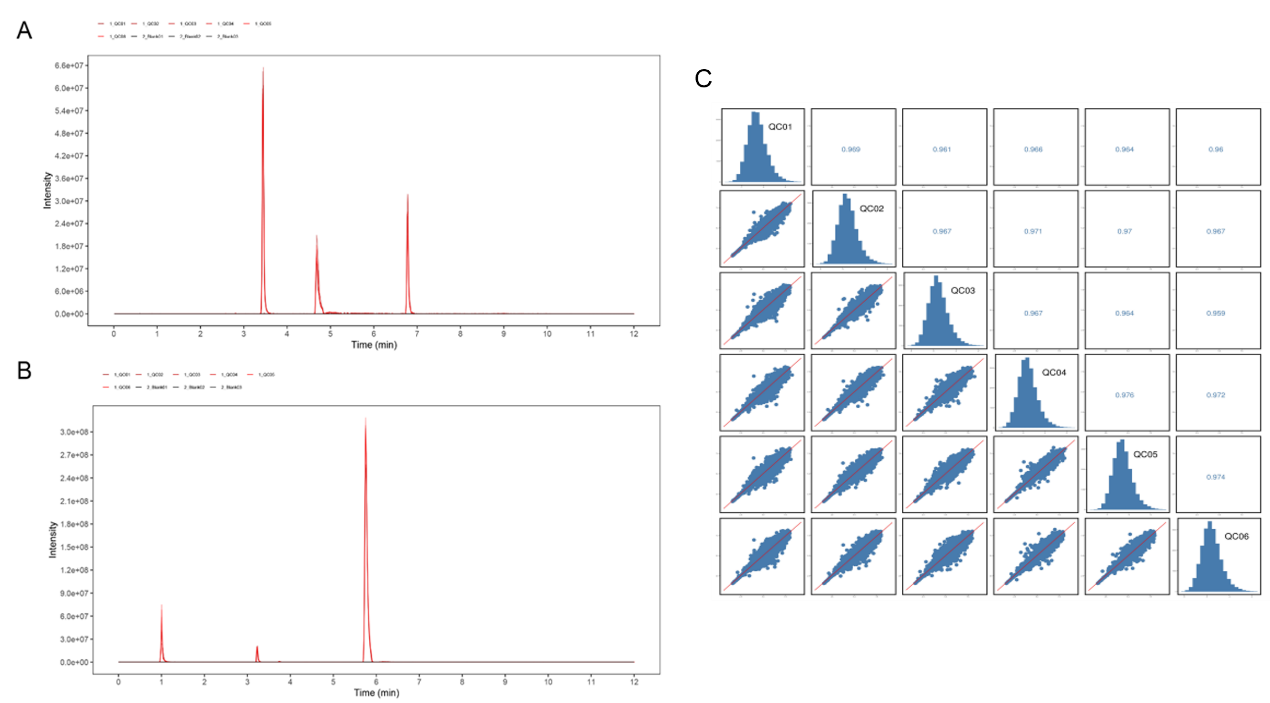


**Figure S4 Quality control results. (A)**: Extracted ion chromatogram of QC samples in POS mode; **(B)**: Extracted ion chromatogram of QC samples in NEG mode; **(C)**: Results of QC sample correlation analysis.


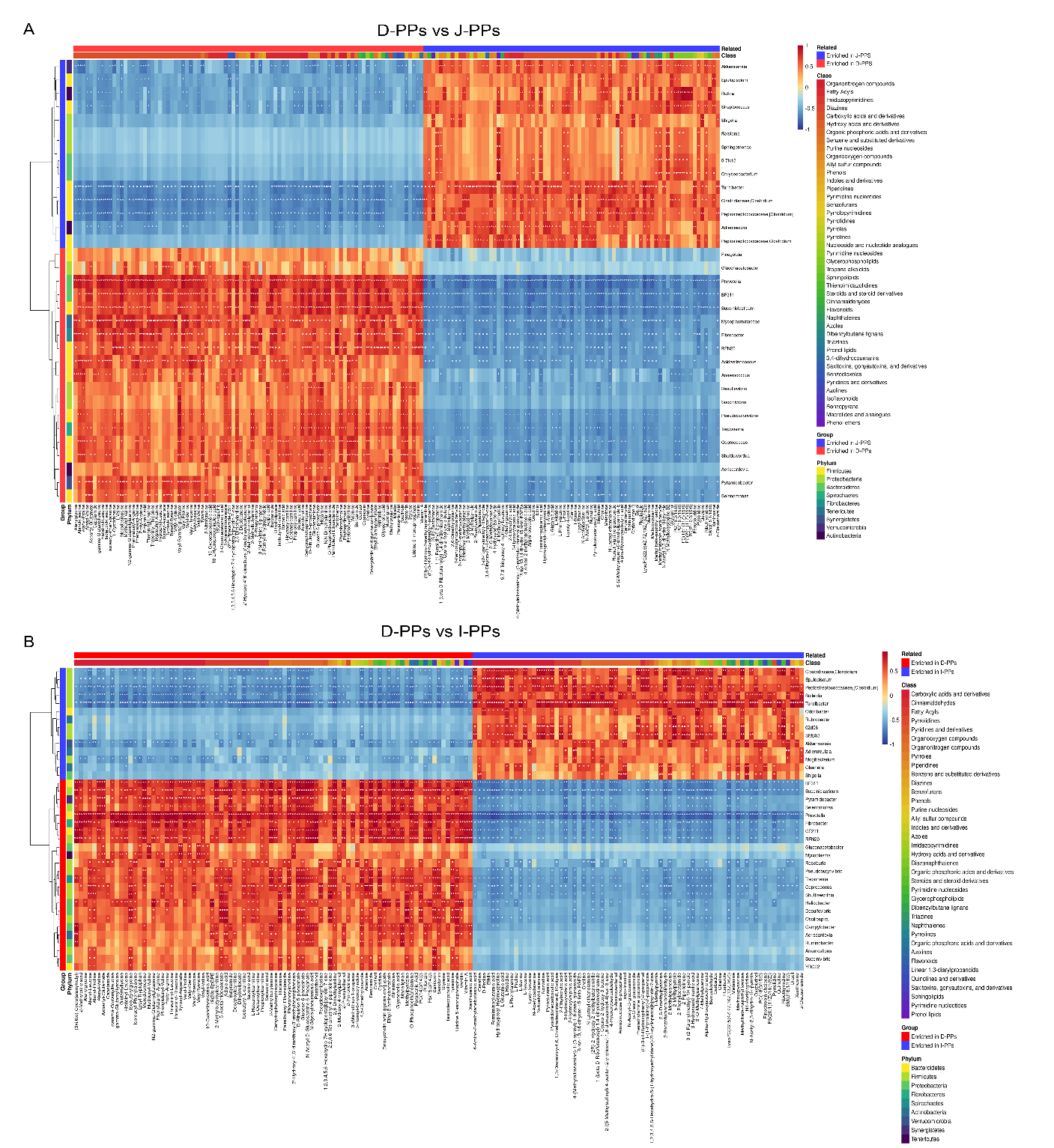


**Figure S5 Integrated correlation analysis of microbiota and metabolites.** Heatmap of the correlation between microbiota with statistical differences at genus level and all differential metabolites. **(A)** Group D-PPs and I-PPs. **(B)** Group D-PPs and J-PPs. The horizontal coordinate shows the differentially abundant metabolites and their classes, while the vertical coordinate shows the differentially abundant bacteria and their phylum classification. Red represents positive correlation, blue represents negative correlation, and white stars represent significant correlation areas (* *P* < 0.05; ***P* < 0.01; ****P* < 0.001).
